# Aesthetics, illusion of success and age interactions: disentangling effects in the closed-loop design with sham neurofeedback training

**DOI:** 10.3389/fnhum.2025.1678940

**Published:** 2025-11-14

**Authors:** Adrian Naas, Scott Rohrbach, Payam Sadeghi Shabestari, Patrick Neff, Andreas Sonderegger

**Affiliations:** 1 Department of Psychology, University of Fribourg/Freiburg, Fribourg, Switzerland; 2 Bern Business School, Institute New Work, Bern, Switzerland; 3 Department of Otorhinolaryngology, Head and Neck Surgery, University Hospital and University of Zurich, Zurich, Switzerland; 4 Department of Psychiatry and Psychotherapy, University of Regensburg, Regensburg, Switzerland

**Keywords:** neurofeedback, sham, aesthetics, non-responder, alpha, workload, motivation

## Abstract

This study examined the influence of the aesthetics of visual feedback stimuli in neurofeedback training (NFB). Previous research shows a lack of specific design standards in NFB research and its application. Beyond limited literature on continuous and intermittent feedback presentation effects, most NFB design parameters remain largely understudied. Studies in the context of interface design has pointed at possible effects of aesthetics and task difficulty, indicating an interaction effect of aesthetics on performance and perseverance in difficult task conditions. The study at hand evaluates whether similar effects emerge in the context of NFB. In order to address this question, aesthetics and NFB illusion of success were manipulated experimentally in a sham NFB study (*N* = 24) following a 2 × 2 within-subjects design. The main dependent variables were perseverance behavior, subjective workload, motivation, and EEG activity. Results indicated an interaction between pleasing design, illusion of success, and participant age affecting perseverance and physical demand. Alpha-1 band amplitudes were modulated by an interaction between pleasing design and age, and a main effect of the illusion of success emerged. Surprisingly, only the illusion of success variable appeared to exert a meaningful influence on the workload and motivation context. Discussing the observed results, the study partially confirms the hypothesis of aesthetics affecting the outcome when the task is difficult in the context of NFB. The relevance of the age variable is addressed, and potential effects in the context of executive functioning and technology adoption processes are considered. Results encourage further research on the topic of NFB design optimization, including verum NFB in the patient population to increase NFB therapy potential.

## 1 Introduction

NFB is a specialized form of biofeedback that utilizes real-time electroencephalography (EEG) or neuroimaging to monitor and modulate cortical activity, enabling individuals to self-regulate neural oscillations impacting cognitive (Nan et al., 2012), emotional (Herwig et al., 2019), and behavioral (Linden et al., 1996) outcomes via feedback-driven adjustments in brain states (Sitaram et al., 2017). NFB has shown positive results in the treatment of various disorders such as Attention Deficit Hyperactivity Disorder (Arns et al., 2014), Autism Spectrum Disorder (van Hoogdalem et al., 2021), Substance Use Disorder (Russo et al., 2023), Epilepsy (Tan et al., 2009), and Tinnitus (Güntensperger et al., 2017), resulting in increased research efforts in recent years. Currently, questions regarding which cortical activity patterns, features, and regions of interest are used, what mental strategies are applied, and how feedback should be provided (e.g., continuous or intermittent) are being explored (Emmert et al., 2017; Oblak et al., 2017). Despite the evidently broad landscape of research efforts, little systematic research has been conducted addressing questions of feedback *design*.

In NFB, information about an individual's brain activity is most commonly presented visually on a computer screen, although auditory, haptic, and electrical stimulation modalities are also employed (Sitaram et al., 2017). Traditional ways to visualize NFB are bar graphs or “thermometers” (Mihara et al., 2021), simple graphics on a screen, like appearance of a sun symbol as stimuli of positive reinforcement (Geladé et al., 2017), as well as floating geometrical shapes like cones and spheres (Schenk et al., 2005). Other NFB applications, particularly in commercial systems, provide feedback within the context of gamified environments. For instance, avatars may exhibit increased speed and accumulate higher scores when the target brain state is successfully attained (Jirayucharoensak et al., 2019). Overall, feedback visualizations are very diverse in the NFB research and practice. To date, no common framework or guideline has been established regarding the design of NFB visualizations (Emmert et al., 2017).

This lack of systematic research in NFB design seems surprising, considering the amount of empirical evidence in the domain of human-computer interaction (HCI) and Human Factors stressing the importance of design aesthetics on user experience and behavior. In this regard, research has shown that design aesthetics positively influence experiential consequences of the interaction with an interface and may increase individuals' motivation and performance (see e.g., Hassenzahl and Monk, 2010; Sonderegger et al., 2012; Sonderegger and Sauer, 2010; Tractinsky et al., 2000; Tuch et al., 2012). In addition, there is some empirical evidence that this positive influence of design aesthetics on motivation, perseverance, and performance is particularly evident in the context of difficult tasks (Moshagen et al., 2009; Reppa and McDougall, 2015).

Considering these findings, it seems plausible that design aesthetics might also affect NFB outcomes via participant motivation, perseverance, and finally, training success. This assumption may be particularly relevant in challenging non-responder scenarios, where individuals fail to exhibit significant improvements in terms of their neural activity patterns. When individuals fail to modulate their brain activity, feedback design might not only influence NFB via a motivational-behavioral pathway but could potentially also have a direct impact on trainees' brainwave patterns. It is, thus, conceivable that specific design patterns influence brain activity (e.g., in the alpha range) and thus conflict with or facilitate the training objective (e.g., the increase of alpha activity). The goal of this study is to explore these two pathways by answering the questions of how NFB aesthetics influence motivation, workload, and behavior, and how EEG activity is influenced by the feedback design. To investigate these questions, an experiment was conducted in which the *aesthetics* of an NFB visualization were systematically manipulated and tested. The implemented sham neurofeedback (S-NFB) procedure enabled the simulation and manipulation of responder and non-responder scenarios in a controlled experimental setting.

### 1.1 NFB

NFB is a learning experience (Alkoby et al., 2018; Haugg et al., 2021) and is comparable to skill acquisition (Sitaram et al., 2017). In the same way that learning variability applies to any learning situation (Shute, 1992), NFB learners sometimes fail to fully succeed in the activity (Davelaar et al., 2018). Indeed, a significant proportion of NFB participants do not have the ability to self-regulate or take control of their cortical activity patterns [e.g., in accordance with (Hanslmayr et al., 2005) up to 50% of participants are unable to influence their brain activity]. In current literature, these participants are commonly referred to as non-responders, non-regulators, or non-performers (Kadosh and Staunton, 2019). The rate of non-responders seems high, and the phenomenon does not only concern the area of NFB. As shown by Vidaurre and Blankertz (2010), 15**–**30% of both male and female users of brain-computer interfaces (BCIs) are unable to exert control, i.e., 15–30% of the general population are assumed to be BCI illiterate.

To address this issue, two methodological pathways have been proposed: The optimization of BCI and NFB protocols (e.g., Lubianiker et al., 2019) and the identification of reliable early markers of non-response (e.g., Alkoby et al., 2018). An exemplary finding in terms of marker identification is that participants showing abnormal brain activity patterns perform better at normalizing their brain-activity patterns than healthy participants aiming to change it (Haugg et al., 2021; Skouras and Scharnowski, 2019). An example in terms of NFB optimization is the process-based NFB training framework by Lubianiker et al. (2019). As *aesthetics* has been shown to influence performance in other contexts (Sonderegger and Sauer, 2010; Moshagen et al., 2009), considering the effects of *pleasing design* might be another pathway of NFB optimization to potentially contribute to a reduction in non-responder rates.

Additionally, while the factors contributing to non-responsiveness remain insufficiently understood, there is a broad consensus that a significant part of the non-responder effect can be explained by psychological factors (Alkoby et al., 2018; Kadosh and Staunton, 2019), and especially motivation seems to play an important role (Alkoby et al., 2018; Kadosh and Staunton, 2019). As learner motivation has been shown to be predictive of successful self-regulation in other domains (see e.g., Slanger et al., 2015), assessing motivational processes in NFB is, thus, critical. It seems plausible that factors like aesthetics might influence trainees' motivation in NFB in situations of duress of non-responsiveness.

### 1.2 Aesthetics

From an interactionist perspective, aesthetics may be conceptualized as an immediate, pleasurable subjective experience elicited in response to the human interaction with a stimulus (Moshagen and Thielsch, 2013). In contrast, *aesthetic properties* of a stimulus describe the characteristics of a stimulus that induce an experience or potentially aesthetic preference (Leder et al., 2004).

According to the Aesthetic Experience Model (Leder et al., 2004), different factors such as contrast, symmetry, and prototypicality influence the aesthetic experience. In addition, research in the domain of human-computer interaction has indicated that characteristics of visual stimuli such as color, balance, structure, order, density, novelty, and complexity are important influencing variables of aesthetic experience (Moshagen and Thielsch, 2010). When applying these principles to the aesthetic experience of NFB stimuli, particular relevance appears to lie in the automatic perceptual processing of stimulus features—complexity, contrast, and symmetry—as well as in the implicit integration of memory-related factors, including familiarity and prototypicality (Leder et al., 2004; Collaud et al., 2022; McDougall and Reppa, 2008).

The aesthetic experience, in turn, influences other variables such as user performance (Sonderegger and Sauer, 2010; Moshagen et al., 2009; Reppa and McDougall, 2015; Moshagen and Thielsch, 2010), learning efficiency (Pomales-García et al., 2005), user engagement (see e.g., Sutcliffe, 2010), user experience (e.g., in terms of usability see Sonderegger and Sauer, 2010), and motivation (Zain et al., 2011; Zhang et al., 2000).

Motivation is defined as “the internal and/or external forces producing the initiation, direction, intensity, and persistence of behavior” (Vallerand and Thill, 1993, p. 18). According to self-determination theory (Ryan and Deci, 2017), motivation is linked to play, exploration, environmental mastery, the emotion of interest, and the novelty and challenges that might prompt interest.” In the context of NFB research, it has been observed repeatedly that motivation is a good predictor of NFB or BCI self-regulation success (see e.g., Nijboer et al., 2008, 2010; Lubianiker et al., 2019, or the reviews by Alkoby et al., 2018; Kadosh and Staunton, 2019). It seems plausible that low motivation and the consecutive lack of finding an appropriate NFB self-regulation strategy create a vicious circle of amotivation (or learned helplessness), creating non-responders in the process. Initial failure to self-regulate might reduce motivation and subsequently impede the search for mental strategies.

Counteracting amotivation effects, aesthetics has been proposed to influence motivational processes and related outcomes such as performance, through mechanisms described by theoretical models (Norman, 2004; Moshagen et al., 2009). The *Positive Affect Mediation Model* (Norman, 2004; Moshagen et al., 2009) suggests that aesthetics lead to positive affect, which in turn increases performance in the context of problem solving. Additional empirical evidence suggests that performance outcomes are affected by motivational processes, which in turn are influenced by aesthetics (Sonderegger and Sauer, 2010).

Moreover, it has been shown that the facilitating effect of aesthetic design on performance is particularly evident in difficult task conditions (Moshagen et al., 2009; Reppa and McDougall, 2015; Nakarada-Kordic and Lobb, 2005). Nakarada-Kordic and Lobb (2005) indicated that more time was spent on aesthetically pleasing web pages compared to the aesthetically non-pleasing web pages, but only when the search condition was difficult compared to the easy search conditions (see also Moshagen et al., 2009, for similar effects of aesthetics on performance in difficult tasks). Similarly, Reppa and McDougall (2015) presented participants with an icon search task, while Moshagen et al. (2009) used a website search task. The findings of both studies converged in demonstrating that the beneficial effect of aesthetic design on task performance emerged exclusively under conditions of a difficult task and when the expectancy of success was low. Referring to the wording of Reppa and McDougall (2015): “When the going gets tough, the beautiful get going.” Enhancing perseverance may represent a viable strategy for addressing the challenge posed by non-responders in therapeutic or intervention contexts.

Elaborating on the effects of aesthetic design, it seems plausible that increasing interface aesthetics to facilitate motivation (Zain et al., 2011), user engagement (Sutcliffe, 2010), and user experience (Sonderegger and Sauer, 2010) could also result in a higher probability of using NFB systems more frequently. Thus, increased adherence in both traditional NFB practice settings and at-home NFB settings (Naas et al., 2025b) could potentially result from increased NFB stimulus aesthetics. Higher NFB frequency (Domingos et al., 2021) combined with higher motivation (Pérez-Elvira et al., 2021) to use the system might lead to more pronounced NFB success (Lubianiker et al., 2019) as “it is well documented that individuals who experience an active interest taken in their performance tend to increase their performance more than do individuals who experience no interest taken in their performance” (Green and Bavelier, 2008).

### 1.3 Neural correlates of pleasing design stimuli

Beyond conventional outcome measures of aesthetics such as task performance and usability, NFB paradigms give rise to the evaluation of neurophysiological effects. The interaction between neural activity patterns and aesthetic stimuli design is of relevance, as aesthetic influences within the closed-loop NFB system may modulate the very neural signatures targeted for regulation. For instance, the orbitofrontal cortex has been shown to respond differentially according to the aesthetic properties of visual stimuli, as evidenced by fMRI studies (Kawabata and Zeki, 2004). Notably, this same region has been successfully targeted in fMRI-based NFB interventions to alleviate symptoms of contamination anxiety (Scheinost et al., 2013). Such findings suggest that aesthetic modulation of neural activity may act as a systematic covariate in the NFB context, warranting the further exploration of aesthetic effects on neurophysiological outcomes.

Apart from these possible covariation effects, neural correlates harbor the potential to indicate whether users are affected by any manipulated variable on a physiological level. Coding typical neural responses to aesthetic or non-aesthetic stimuli is the core concept of the emerging neuroaesthetics field (Ramachandran and Hirstein, 1999). As NFB usually comprises mundane stimuli and designs, it is vital to discuss the neuroaesthetic correlates of such stimuli. Indeed, a specific blood flow response identifying relevant areas in the frontal, temporal, cingulate, and tempoparietal cortex has been found in response to aesthetically more pleasing stimuli (e.g., in the context of visual textures and geometrical shapes, Jacobsen et al., 2006). Other studies have identified the orbito-frontal cortex and medial-frontal cortex, the ventral striatum, the anterior cingulate cortex, and the insula, which respond to aesthetically pleasing visual stimuli with increased fMRI activity (Kawabata and Zeki, 2004; Jacobsen et al., 2006; Jacobs et al., 2012; Vartanian and Goel, 2004). Above and beyond the mentioned structures, there are also results showing aesthetic stimuli like paintings and photographs show specific increases in terms of MEG and fMRI activity in the dlPFC (Cela-Conde et al., 2004) and the mOFC (Ishizu and Zeki, 2011).

The presented studies (Kawabata and Zeki, 2004; Jacobsen et al., 2006; Jacobs et al., 2012; Vartanian and Goel, 2004; Ishizu and Zeki, 2011) have established a link between fMRI activity and aesthetic perception. Caution is warranted when neuroaesthetics effects from fMRI research are transferred to the context of EEG NFB. However, empirical findings (Hanslmayr et al., 2011) indicate a relationship between fMRI BOLD signals and EEG alpha activity, with studies reporting this association to be predominantly inverse in nature (Laufs et al., 2003). EEG NFB is currently one of the primary types of NFB (Fathi et al., 2025). Visual design aspects influencing the very brain activity pattern that is the target of NFB paradigms (for example, EEG beta activity in the context of beta/theta NFB) would have to be taken into consideration as a covariate. In the current NFB research literature, no such efforts are undertaken, and no knowledge about the possible, systematic aesthetic effects of feedback stimuli is available.

The neuroaesthetic effects complement previous research in the domain of human-computer interaction, showing that design can have a major influence on attitudes, emotions, and behavior (Sonderegger and Sauer, 2010; Tractinsky et al., 2000; Hekkert and Leder, 2008; Thielsch et al., 2019). Neither perspective (neuroaesthetic research or user experience research) has, to this day, evaluated possible effects of NFB design on the trainee systematically. It is plausible that the specific properties of the feedback implementation (e.g., color, speed of change in scenery, specific feedback signal displayed as change in position, color, or shape) have an inert effect on the trainee.

Establishing the link between aesthetic perception and brain activity gives rise to the question of how design aesthetics influences NFB users above and beyond NFB training effects. In the context of design research, this notion is often discussed in terms of the importance of the medium as opposed to the content of the message (McLuhan and Fiore, 1967). Thus, it seems crucial to consider not only *what* information is transmitted to the user but *how* it is transmitted (Reppa and McDougall, 2015; McLuhan and Fiore, 1967). Or, putting it differently, in light of the discussed neuroaesthetic effects (e.g., Kawabata and Zeki, 2004; Cela-Conde et al., 2004; Ishizu and Zeki, 2011; Chatterjee et al., 2009; Handy et al., 2010), it remains unknown how inherent NFB effects are helped or counteracted by differences in NFB visualization aesthetics. Applying results from neuroaesthetic research allows us to identify potential brain regions involved in the process of implicit aesthetic effects in NFB users. In this regard, especially the fusiform face area (FFA; Chatterjee et al., 2009) as well as medially close areas and the reward circuitry (Kim et al., 2007; Kühn and Gallinat, 2012; Winston et al., 2007) seem to be implicated in the process, indicating frontal areas as potential regions of interest for effects of aesthetics in the context of NFB.

Effects of automatic aesthetic judgment have been observed not only with different types of stimulus material like faces (Chatterjee et al., 2009), music (Blood and Zatorre, 2001), logos (Handy et al., 2010), and webpages (Lindgaard et al., 2006) but also emerge through the lens of different imaging techniques like fMRI and EEG (Belfi et al., 2019; Vessel et al., 2012). The lack of literature on the topic of visual aesthetic effects on neural correlates is surprising, as the visual aesthetic experience might directly and systematically influence NFB target outcomes. To date, the covariate effects of aesthetic design on cortical activity patterns (e.g., alpha) cannot be excluded in the closed-loop system, and it is crucial to enhance the understanding of the underlying effects of NFB aesthetics.

### 1.4 The present study

The goal of the study at hand is two-fold. First, the effects of *aesthetics* on objective and subjective outcome variables of NFB are evaluated in the *non-responder* context. Note, this study manipulates NFB non-responders with an S-NFB procedure, giving participants the *illusion of success* or *no success* (see Section 2.2). Second, the sham feedback design allows for disentangling NFB effects from effects inherent to the NFB design on the neurophysiological level.

The presented literature on learning, usability, and performance shows that workload, motivation, and perseverance differ according to *pleasing design* and task *success* (Bauer et al., 2016). In accordance with the presented literature on *aesthetics, motivation*, and *perseverance* (Sonderegger and Sauer, 2010; Nakarada-Kordic and Lobb, 2005), hypothesis **H1.1** suggests that: *Motivation scores and perseverance are higher for the aesthetically pleasing S-NFB design than for the aesthetically non-pleasing S-NFB design*. Based on the discussed research on aesthetic design and workload (Sonderegger and Sauer, 2010; Miller, 2011), hypothesis **H1.2** suggests that: *Perceived workload is lower for aesthetically pleasing S-NFB interventions than for aesthetically non-pleasing S-NFB interventions*.

In line with the introduced results on self-regulation, *motivation*, and *perseverance* (Kadosh and Staunton, 2019; Halperin and Eldar Regev, 2021), hypothesis **H2.1** suggests that: *Illusion of success during an S-NFB intervention increases motivation and perseverance times compared to a no success condition*. In concordance with the addressed findings on task *success* and *workload* (Kadosh and Staunton, 2019), hypothesis **H2.2** is generated: *Illusion of success during an S-NFB intervention decreases workload in comparison to a no success condition*.

The main hypothesis of this study relates to a potential interaction effect between *aesthetics* and the *illusion of success*. According to studies by Reppa and McDougall (2015) and Moshagen et al. (2009), a positive effect of *aesthetics* on *performance* is observable when the task is difficult. In line with this effect pattern, we expect that *pleasing design* positively affects *perseverance, workload*, and *motivation* in NFB, especially when the task is difficult (i.e., in the condition of *no success*). We expect (**H3**) that *the positive effects of pleasing design on motivation, perseverance, and the negative effects of pleasing design on workload are especially relevant in the no success condition*.

Finally, in accordance with the presented neuroaesthetic effects (Handy et al., 2010; Kühn and Gallinat, 2012; Winston et al., 2007; Lindgaard et al., 2006; Blood and Zatorre, 2001; Lebreton et al., 2009), the following research question is formulated: Does *pleasing design* and/or *illusion of success* systematically influence brain activity patterns that are used in the context of NFB (e.g., alpha amplitudes)? Given the limited empirical evidence on EEG spectral analyses in responses to aesthetics, we abstain from formulating a directed hypothesis. In accordance with the presented literature on neural correlates of the perception of *aesthetic* stimuli, the hypothesis **H4** is formed (Kawabata and Zeki, 2004; Jacobsen et al., 2006; Jacobs et al., 2012; Cela-Conde et al., 2004; Ishizu and Zeki, 2011; Kim et al., 2007; Chatterjee et al., 2009): *The S-NFB manipulations (pleasing design and illusion of success) affect alpha spectral power*.

## 2 Materials and methods

### 2.1 Participants

Participants were recruited via private mailing lists and advertising on campus. After receiving comprehensive information about the study protocol, all participants provided their written informed consent. A total number of 24 participants ranging in age between 20 and 37 years (*M* = 23.83 years, *SD* = 3.56 years, 6 women) took part in the study.

The majority of the participants (*n* = 12) had attained a high school diploma as their highest level of education, *n* = 7 participants had a university degree, *n* = 2 participants had completed a higher education course, *n* = 2 had finished an apprenticeship, and *n* = 1 had completed primary school. All participants were native French speakers, had no psychiatric or neurological disorders, and had normal or corrected-to-normal vision. Additionally, none of the participants had any prior experience with NFB. As compensation, psychology students received course credits through the university's intern credit system. Non-psychology student participants received a bar of chocolate. This study received ethical approval from the internal review board of the Department of Psychology at the University of Fribourg (reference No. 2022-802 R1).

### 2.2 Experimental design

**Figure 1:**
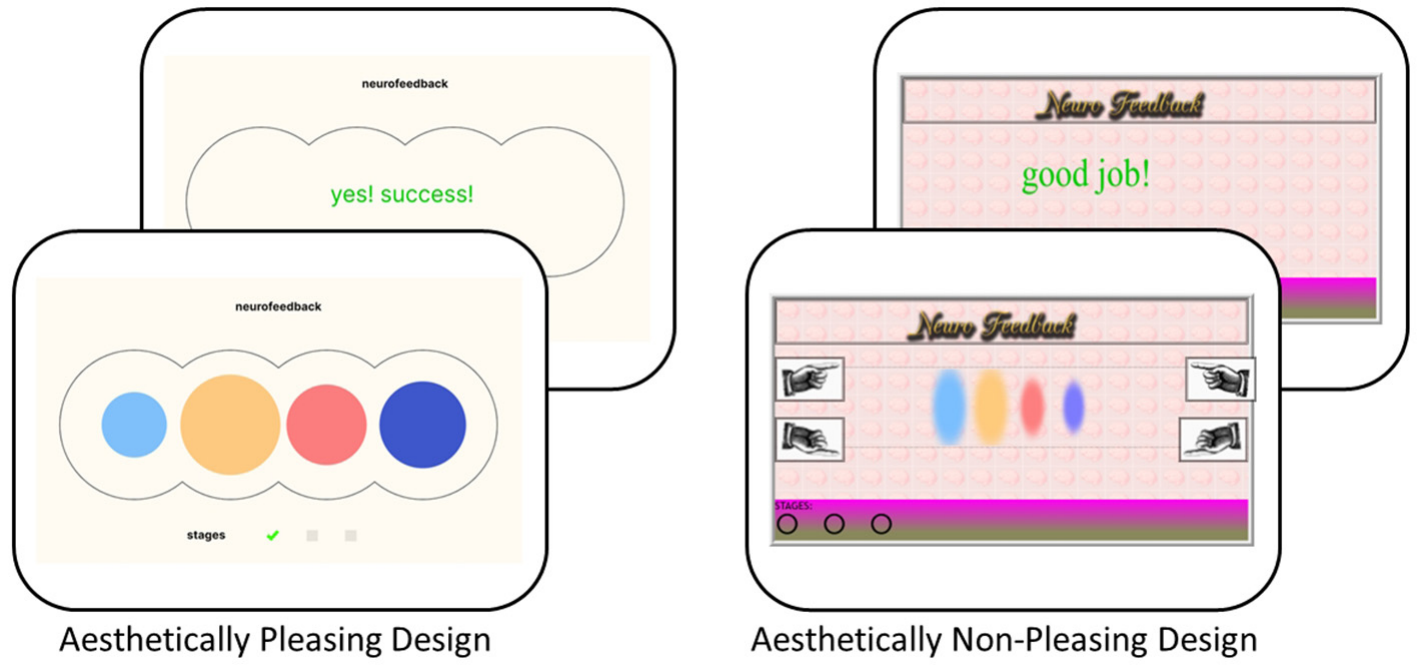
Display of two aesthetics conditions: “pleasing design” and “non-pleasing design.” In both *aesthetic* conditions, participants were advised to apply a mental strategy that increased the size of the spheres. In order to achieve that goal, participants were instructed to “let the feedback guide” them.

A 2 × 2 experimental design was applied, with *aesthetics* (*pleasing design* vs. *non-pleasing design*, see Figure 1) and *illusion of success* (*success* vs. *no success*) manipulated as within-subjects predictors. The repeated measures design of the study at hand was chosen to account for interindividual differences in participants' reactions to the S-NFB intervention. All participants completed all combinations of the 2 × 2 research design in counterbalanced order, following a randomized block design. In the *non-pleasing* condition, the feedback texts were replaced with different wordings: “good job!” in green font and a yellow smiley or “uh oh… try again…” in red text color with a yellow frowning face.

**Figure 2:**
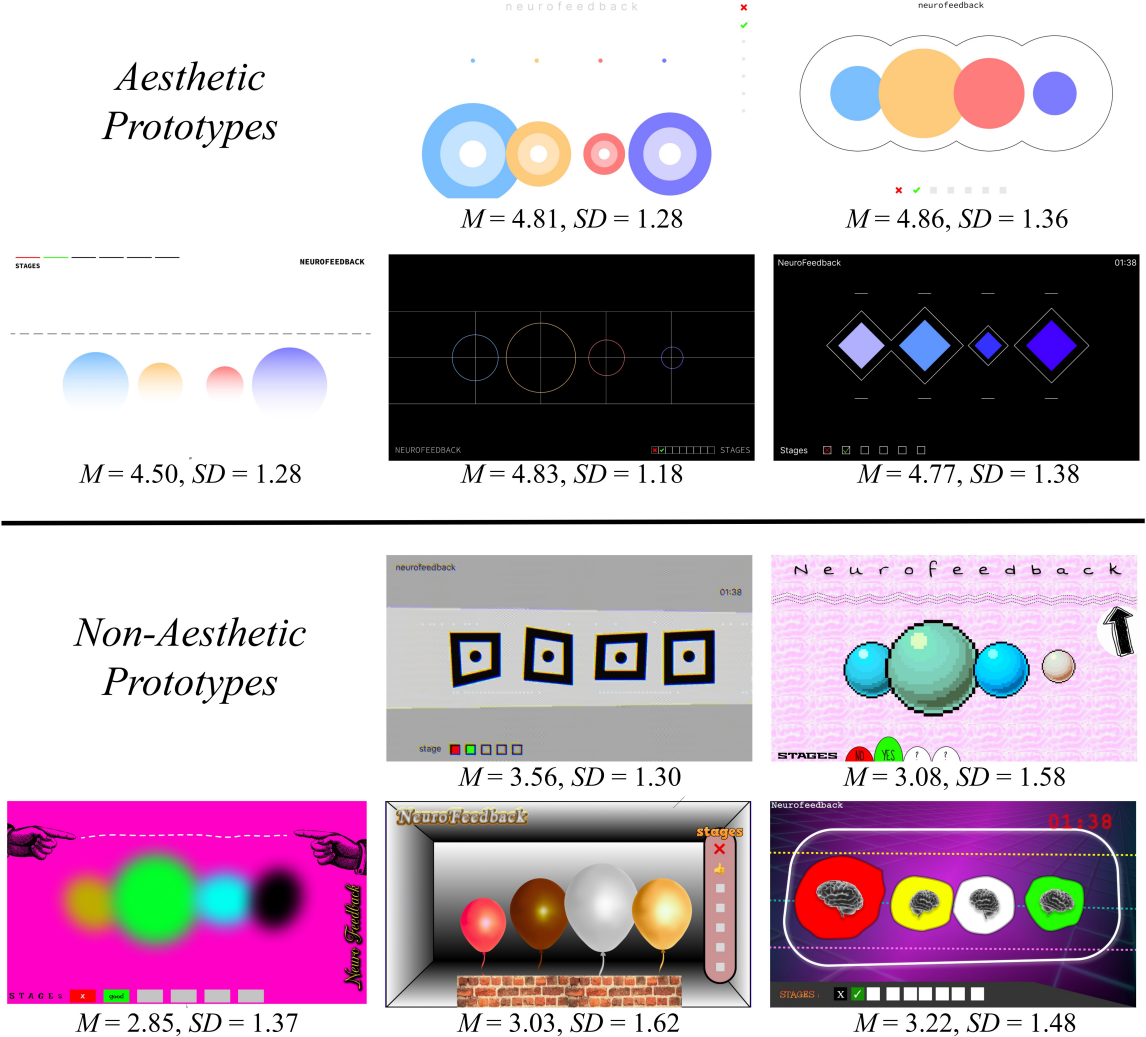
Pleasing and non-pleasing pretest prototypes. Aesthetics were assessed by means of the VisAWI short scale, and respective sum score means (*M*) and standard deviations (*SD*) can be derived from the figure. In the pretest, task difficulty was not manipulated.

The pleasing and non-pleasing designs were derived from a pre-study (*N* = 60 participants, see Figure 2). The within-subjects pre-study compared five *pleasing* and five *non-pleasing* visualizations and showed differences regarding their visual attractiveness measured by the Visual Aesthetics of Websites Inventory short scale (VisAWI; Moshagen and Thielsch, 2010); *F*_(1, 59)_ = 108.11, *p* ≤ 0.001, $\eta_{\text{p}}^{2}$ = 0.95. The prototypes used in the present study (see Figure 1) represent a further developed and refined version of the two prototypes that obtained the highest and lowest aesthetics ratings in the pre-study.

To implement the sham character of the main study, the second predictor, illusion of success, was introduced with two levels: success and no success. In the context of NFB, a “sham” condition refers to “feedback [that is] not derived from the participant's brain activity” (Thibault et al., 2016). The sham neurofeedback (S-NFB) display—such as incremental increases in circle size—included stochastic variation designed to enhance the plausibility of the NFB procedure. In the success condition, the S-NFB presented a pre-programmed linear increase over time superimposed with random fluctuations, thereby simulating apparent self-regulation success. Conversely, in the no success condition, the feedback signal exhibited only random fluctuations without any directional trend, creating the impression of unsuccessful regulation attempts. Additionally, the system created positive messages (green font: “yes! success!”) and negative feedback (red font: “not successful”).

The dimensional indicator of illusion of success was circle size (see Figure 1), and it was set to an average of 50.33% over the duration of the 3 min of obligatory NFB in the success condition. In the no success condition, the respective average circle size was set to 11.76%. Moreover, participants received two types of categorical feedback. Firstly, the circles could reach maximum size. In the success condition, during 11.76% of the time, at least one of the circles reached maximum circumference. In the no success condition, the circles reached maximum circumference 0% of the time. Secondly, in the success condition, participants always succeeded in passing the stages, while in the no success condition, participants never passed the stages.

### 2.3 Measures and instruments

All items of the measuring instruments that were custom-made are presented in the supplementary materials (Naas et al., 2025a). The order of the items remained the same across all conditions and for all participants. For scales that had to be translated from English to French, the back-translation procedure was applied. One member of the team translated the questionnaires from English to French, and another member of the team translated the created French version back to English. A third member of the team compared the original English version with the back-translated English version of the questionnaire and judged their similarity. Then, differences were discussed, and the French version was adapted until all translators were satisfied with the solution.

To judge the manipulation quality of aesthetics by means of the S-NFB stimuli, the VisAWI short scale was used as a manipulation check (Moshagen and Thielsch, 2010). The VisAWI comprises seven-point Likert scales ranging from 1 “strongly disagree” to 7 “strongly agree.” The scales' observed internal consistency in the current study varied between poor and excellent from timepoints T1–T4 (Cronbach's α range = 0.59–0.93).

In order to measure participants' motivational reaction to visual feedback, the Situational Motivation Scale was applied (SIMS; Guay et al., 2000). The self-report scale assesses intrinsic motivation, identified regulation, external regulation, and amotivation. Only the global values of the scale were considered in this study, reducing participant burden by applying only the subscales *intrinsic motivation* and *amotivation*. Participants rated the four items of each construct on a seven-point Likert scale (1 = “I do not agree at all,” 7 = “I fully agree”). The order of items corresponded to the order published in Guay et al. (2000). The observed internal consistency for the subscale of *intrinsic motivation* varied between acceptable and good (Cronbach's α range = 0.73–0.86). Internal consistencies for the *amotivation* subscale varied between good and excellent (Cronbach's α range = 0.82–0.92).

The NASA-Task Load Index (NASA-TLX) developed by Hart and Staveland (1998) was used to measure participants' workload. The NASA-TLX is a multidimensional self-assessment scale with six subscales, each measuring one dimension: Mental demand, physical demand, time demand, frustration, effort, and performance. The subdimensions Mental Demand and Physical Demand were chosen for further investigation as the item wording corresponded most closely to the task participants performed during S-NFB. Subscales comprise a single item assessed with a scale ranging from 1 to 100. The overall scale ranges from 1 (very low) to 100 (very high). Average values of the raw overall scores of the six items were used to estimate the overall subjective workload (Hart, 2006). The test-retest reliability of the NASA-TLX reported by Battiste and Bortolussi (1988) can be considered acceptable (*r* = 0.77), and its validity is superior to other instruments measuring subjective workload (Hoonakker et al., 2011). As the different subscales of the NASA-TLX rather constitute different constructs per scale, no indicators of internal consistencies were calculated with the data of this study.

Perseverance is defined as “the state of […] keeping at a task […] despite the […] discouragement or the effort involved” (APA, 2018, Persistence section, para. 2). The construct was measured behaviorally via duration (s) of time the participant engaged in the second, voluntary part of S-NFB sessions. Time on task has been used in other studies as a perseverance measure, e.g., by Eaton and Tieber (2017). Note, as perseverance can be classified as part of the executive functions (Ferguson et al., 2021), it is supported by prefrontal cortex activity (Ball et al., 2011; Kolk and Rakic, 2022) and is known to vary with age; participant age was recorded during data collection.

### 2.4 Materials and equipment

During the experiment, two computers were used for data acquisition. A 13-inch MacBook Air (early 2015), version 10.15.7 with a resolution of 1440 × 900 pixels and a screen refresh rate of 60 Hz, was set up to display the visualization of the S-NFB as well as the questionnaires. The second computer, an Intel^®^ Core™ i5-6300U CPU with Windows 10, a resolution of 1920 × 1080, and a screen refresh rate of 60 Hz, was used to process EEG recordings. The questionnaire was programmed on the online survey software Unipark (https://www.unipark.com/), version EFS 21.2.

The S-NFB software, specially developed for this study, was a web application made by Luca Sassoli De Bianchi of EPFL + ECAL Lab (https://as-nefe.firebaseapp.com/). For the measurement of the perseverance variable, a stopwatch was used. Information on the *illusion of success* manipulation can be found in the Section 2.2.

Participants' EEG activity was recorded using a low-impedance electrode gel (ECI Electrode Gel) and a small (50–54 cm), medium (54–58 cm), or large (58–62 cm) 21-channel OpenBCI EEG cap with proprietary, sintered Ag/AgCl electrodes. The cap was connected via 1.5 mm Touch-Proof connectors to the 16-channel (+ reference and ground) OpenBCI Cyton + Daisy PCBs. Both of the boards include eight high-gain and low-noise input channels (OpenBCI, 2022), comprise a 24-bit channel data resolution, and can be connected to passive as well as active electrodes. The boards encompass the Texas Instruments ADS1299 ADC. The channel inputs are designed to handle voltages between ~3.3 and 12 V, and in the current study, a 4-AA battery pack provided power for operation. The RFduino Low Power Bluetooth radio transmitted the data to the Bluetooth dongle. The dongle includes the RFD22301 radio module from RFdigital and an FT231X USB-to-serial converter from FTDI. Sampling rate was set to 125 Hz. The OpenBCI graphical user interface recorded the data into text files that were stored on a local hard drive for subsequent data analyses.

Electrode sites were chosen according to the international 10–20 placement system: FP1, FP2, AFz, F3, F4, F7, F8, Fz, Cz, C3, C4, T7, T8, P3, P4, O1, O2, and CPz. CPz was the reference electrode, and ground was assigned to AFz. To ensure contact quality, the scalp at each of the electrode sites was cleaned with a cotton swab and alcohol before conductive gel was administered. Impedances were kept below 20 kOhm at all times. Regions of interest were the orbito-frontal cortex (OFC) comprising BAs 10, 11, and 47, which were measured with electrodes FP1, FP2, F7, and F8. The medial frontal cortex (MFC), consisting of BAs 9, 10, 11, 12, and 25, was covered with electrodes Fp1, Fp2, F3, and F4. The dorso-lateral prefrontal cortex (dlPFC) is located at BAs 9 and 46, both of which can be captured by means of electrode sites Fp1, Fp2, F3, F4, F7, and F8.

### 2.5 Procedures

Participants were tested at the University of Fribourg, and inclusion and exclusion criteria were checked at the beginning of the experiment. The general study topic was introduced (visual design in the context of NFB), and all participants' questions were answered. Participants were subsequently reminded of their right to discontinue participation at any point without the obligation to provide a reason. Participants signed the informed consent form and continued with the socio-demographic questionnaires (see Supplementary material 1, Naas et al., 2025a).

**Figure 3:**
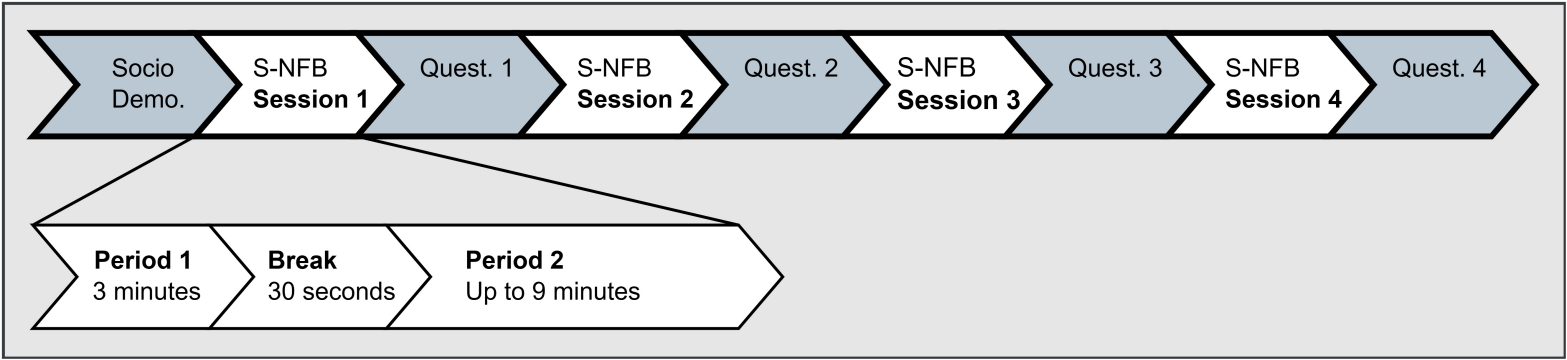
Timeline of the S-NFB experiment procedures.

The EEG was applied, and participants were reminded of the non-invasive nature of EEG measurements. When impedance measures below 20 kOhm were achieved, a 3-min eyes-open EEG baseline measurement was carried out during which participants were advised to look at a fixation cross and to blink as little as possible to avoid eye movements and blink artifacts. The main part of the experiment consisted of four S-NFB sessions corresponding to the four experimental conditions. Each S-NFB session consisted of an initial fixed period of 3 min, followed by an optional second period ranging from 0 to 9 min, the duration of which was determined by the participant. The voluntary second period length indicated participants' perseverance. After each S-NFB session, visual aesthetics (VisAWI short scale, Moshagen and Thielsch, 2013), motivation (SIMS; Guay et al., 2000), and workload (NASA-TLX; Hart and Staveland, 1998) were assessed (see Figure 3).

In the context of the **S-NFB task**, participants were asked to imagine themselves in a calm and peaceful state. It was explained that the four oval shapes grew over time if the participants decreased their mental agitation (Naas et al., 2025a). In relation to the S-NFB illusion, participants were instructed to “make the four oval shapes grow as large as possible” (see Supplementary material, Naas et al., 2025a). Participants were led to believe they were successful in the task of “calming the mind” during two sessions and not successful during two other sessions (predictor *illusion of success*). Moreover, the conditions, *pleasing design* vs. *non-pleasing design*, were varied within subjects. Note, the order of trials was counterbalanced using a randomized block design, minimizing potential carry-over effects. Detailed information on the S-NFB task can be found in the Section 2.2.

When the experimental procedure ended, the EEG cap was removed, a towel and shower gel were provided, and participants washed their hair in the nearby washing facilities. Finally, participants were debriefed and compensated for their efforts (see Section 2.1). Overall, the experiment lasted 90 min, starting times ranged between 06:44 a.m. and 04:15 p.m. (*M*_*StartingTime*_ = 10h19, *SD*_*StartingTime*_ = 02h41).

### 2.6 Statistical analysis and preprocessing

An a priori power analysis was carried out with G^*^power version 3.1.9.7 (Faul et al., 2007). Assuming large effects (i.e., *f* = 0.40) for the interaction of *aesthetics* and *illusion of success*, similar to the ones reported in Reppa and McDougall (2015), setting the alpha error probability to 0.05, 1-beta to 0.80, the correlation among repeated measures at an estimated 0.5, and non-sphericity correction ε to 0.75, the estimated sample size was 16. In order to account for potential measurement issues and to be able to identify smaller effects, the planned sample was increased by 50%, resulting in a final number of 24 participants. Setting alpha error probability to 0.05 for all analyses offered a pragmatic compromise between minimizing the risk of Type I errors and maintaining sufficient statistical power to detect true effects, while keeping sample size requirements and study costs at a manageable level. Analyses were performed using R Studio version 4.5.1 (RStudio Team, 2020).

The following general procedure was chosen: For all analyses, a target multilevel linear model was created by means of the r-package “lme4” (Bates et al., 2015) and R (R Core Team, 2024). The general model was defined as *Outcome* ~ *Aesthetics* ^*^ *IllusionOfSuccess* ^*^ *Age* + *(1|ID)*. All the respective models were compared to a corresponding model 0, which was defined as *Outcome* ~ *1* + *(1|ID)*. The improvement of fit was analyzed and reported by means of an ANOVA. In the case of a non-satisfactory fit, interaction terms were eliminated iteratively in a data-driven, hierarchy-respecting procedure by means of the *step()* function of the “lmerTest” package. The final model was chosen in accordance with the best (lowest) AIC and was compared to model 0 to report goodness of fit indices. Then, estimated marginal means plots were created for significant effects. Note, *age* was centered around the grand mean to provide fixed effect estimates interpretable at the typical age of the sample.

To infer potential *exploratory*, directed connections between the experiment's predictor and outcome variables, a Bayesian network approach was applied. The structure was learned using a constrained hill-climbing algorithm from “bnlearn” (Scutari, 2010) package, with arcs estimated using pairwise Pearson correlations. The resulting directed acyclic graph (DAG) reflects possible directional dependencies among neurophysiological (EEG), behavioral, subjective, and demographic variables. As a model robustness check, additionally, the Tabu search algorithm was applied by means of “bnlearn.” The algorithm is a score-based structure learning method that uses a local search with memory (tabu list) to avoid cycling and local optima (Glover, 1986). The algorithm iteratively explores network structures by accepting non-improving moves temporarily, thereby enhancing global search efficiency.

The collected EEG data in this study were preprocessed by means of a custom-made Matlab 2018a (MathWorks, 2018) pipeline using the plugins EEGLAB (Delorme and Makeig, 2004) and ERPLAB (Lopez-Calderon and Luck, 2014). Preprocessing included the application of the EEGLAB default FIR bandpass filter (*pop_eegfiltnew*) with cutoff frequencies between 1 and 30 Hz. The procedure effectively attenuated power-line noise in the data. As a result, no additional notch filtering was deemed necessary. This decision was supported by visual inspection of the raw EEG traces and corresponding power spectral density plots, which confirmed the absence of residual line noise.

Hamming window type was applied, and the order of the filter was set to the automatic default. In default mode, the filter order and transition bandwidth were estimated using a heuristic procedure. For the applied bandpass filter, the transition bandwidth was set to 25% of the lower passband edge frequency, with a minimum value of 2 Hz to ensure numerical stability. If this criterion could not be met—typically due to the proximity of the passband to a critical frequency (i.e., 0 Hz or the Nyquist frequency, defined as half the sampling rate)—the transition bandwidth was instead determined by the available distance between the bandpass edge and the nearest critical frequency. This approach ensured a feasible filter design whilst minimizing spectral distortion and edge effects.

Then, the data were epoched into sections according to the five experimental conditions (baseline, condition 1, condition 2, condition 3, condition 4). Afterwards, the data were partitioned into a series of non-overlapping one-second epochs for subsequent artifact rejection and processing by means of *eeg_regepochs()*. Following segmentation, artifact-prone epochs were automatically detected and removed via *pop_autorej()*, which applies iterative *SD*-based thresholding to identify epochs with extreme or improbable values. Finally, mean amplitudes of alpha frequency bands were calculated with the function of *pop_fourieeg()*. Individual alpha amplitudes were calculated by, firstly, identifying the individual alpha peak (IAP) frequency between 7.5 and 12.5 Hz. Secondly, the *alpha-1* (lower alpha) amplitudes were derived by setting the cut-off frequencies between IAP**—**2 Hz and IAP. The frequency range between IAP and IAP + 2 Hz (Klimesch et al., 1999) was used to compute *alpha-2* (upper alpha) amplitudes.

## 3 Results

### 3.1 Manipulation check

To assess whether the experimental manipulation of aesthetics was successful, a simple mixed model was used: *VisAWIGlobal* ~ *Aesthetics* + *(1|ID)*. The *pleasing design* conditions scored higher (*M* = 5.05, *SD* = 1.37) than the *non-pleasing* design conditions (*M* = 3.48, *SD* = 1.31), the difference was significant, *t*_(71)_ = 7.84, *p* < 0.001. Thus, the results confirmed a successful manipulation of *aesthetics*. Similarly, in order to check the manipulation of *illusion of success*, a model defined as *Performance* ~ *IllusionOfSuccess* + *(1|ID)* and the *success* conditions (*M* = 65.52, *SD* = 20.19) showed higher performance ratings than the *no success* conditions (*M* = 31.56, *SD* = 28.04), the difference was significant;*t*_(71)_ = 6.92, *p* < 0.001.

### 3.2 Perseverance

**Figure 4:**
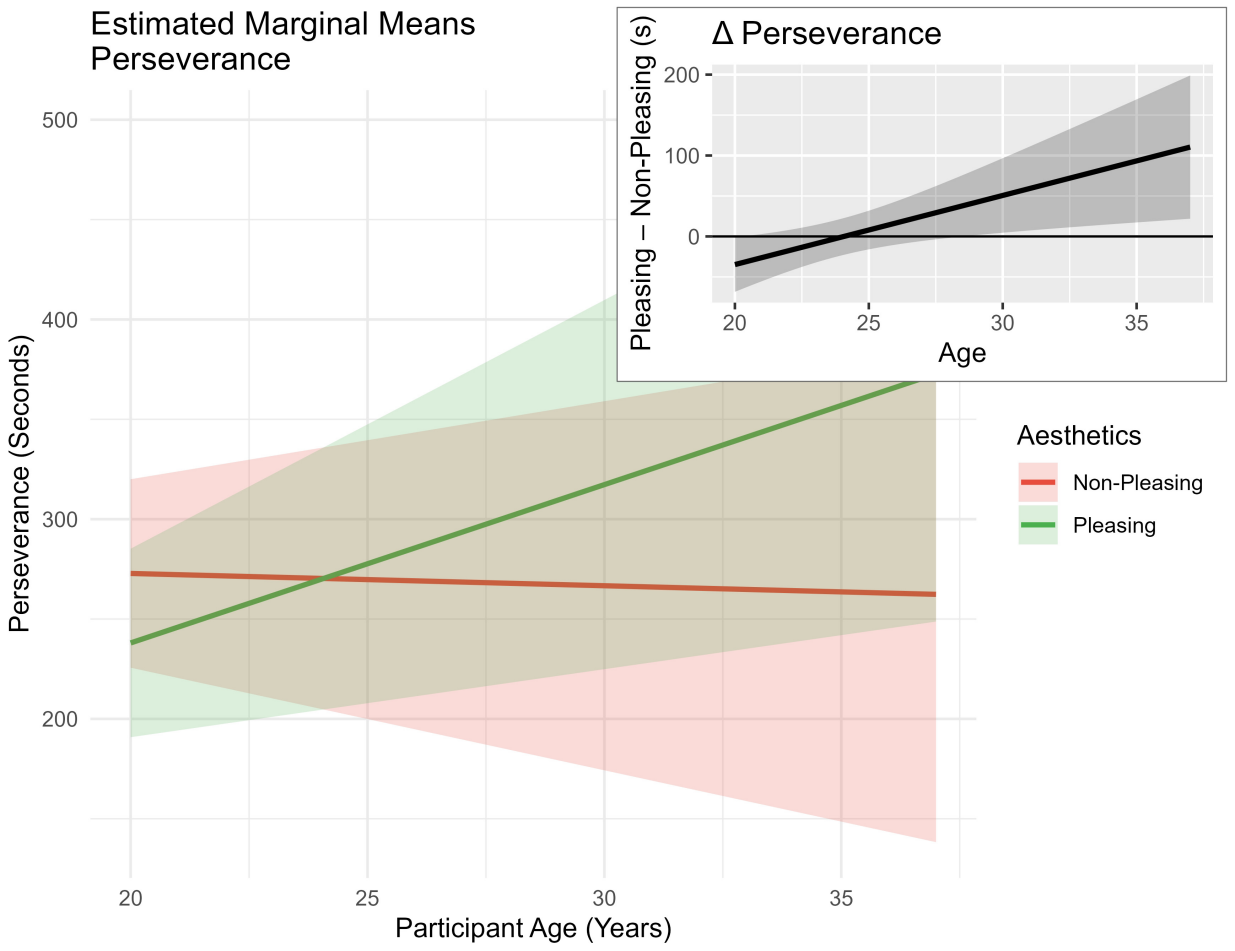
Estimated marginal means of “perseverance” predicted by “aesthetics” and “age.” Based on the mixed model. *Age* was centered with the sample mean. Shaded areas represent ±1 standard error of the mean (SEM).

A mixed model was calculated for the analyses of perseverance with the model *Time*_*Perseverance*_ ~ *Aesthetics* ^*^ *IllusionOfSuccess* ^*^ *Age* + *(1|ID)*. Results showed the model improved fit compared to model 0, $X_{\left(7,\text{N}=24\right)}^{2}$ = 16.03, *p* = 0.025. Results (see Table 1) showed a positive interaction effect between *age* and *pleasing design*, indicating that the *perseverance* effect of *pleasing design* may increase with *age* (see Figure 4).

**Table 1:** Mixed-model analyses regression: effects of predictors “aesthetics,” “illusion of success,” and “age” on “perseverance.”

**Predictor**	**Estimate**	**Std. Error**	***df***	***t-*value**	***p***	*** $\eta_{\text{p}}^{2}$ ***
(Intercept)	276.08	35.55	42.75	7.766	**< 0.001**	**–**
PleasDes	−39.96	32.03	66.00	−1.247	0.217	0.000
Succ	−11.25	32.03	66.00	−0.351	0.727	0.021
Age	0.67	10.20	42.75	0.066	0.948	0.008
PleasDes:Succ	75.83	45.30	66.00	1.674	0.099	0.041
PleasDes: Age	21.96	9.19	66.00	2.388	**0.020**	0.025
Succ: Age	−2.58	9.19	66.00	−0.280	0.780	0.084
PleasDes:Succ: Age	−26.82	13.00	66.00	−2.063	**0.043**	0.061

The table summarizes the statistical analyses of predictors of illusion of success (success vs. no success), aesthetics (pleasing design vs. non-pleasing design), and age (centered) on **perseverance**. PleasDes, pleasing design; Succ, success; df, degrees of freedom; p, probability of error.

**Figure 5:**
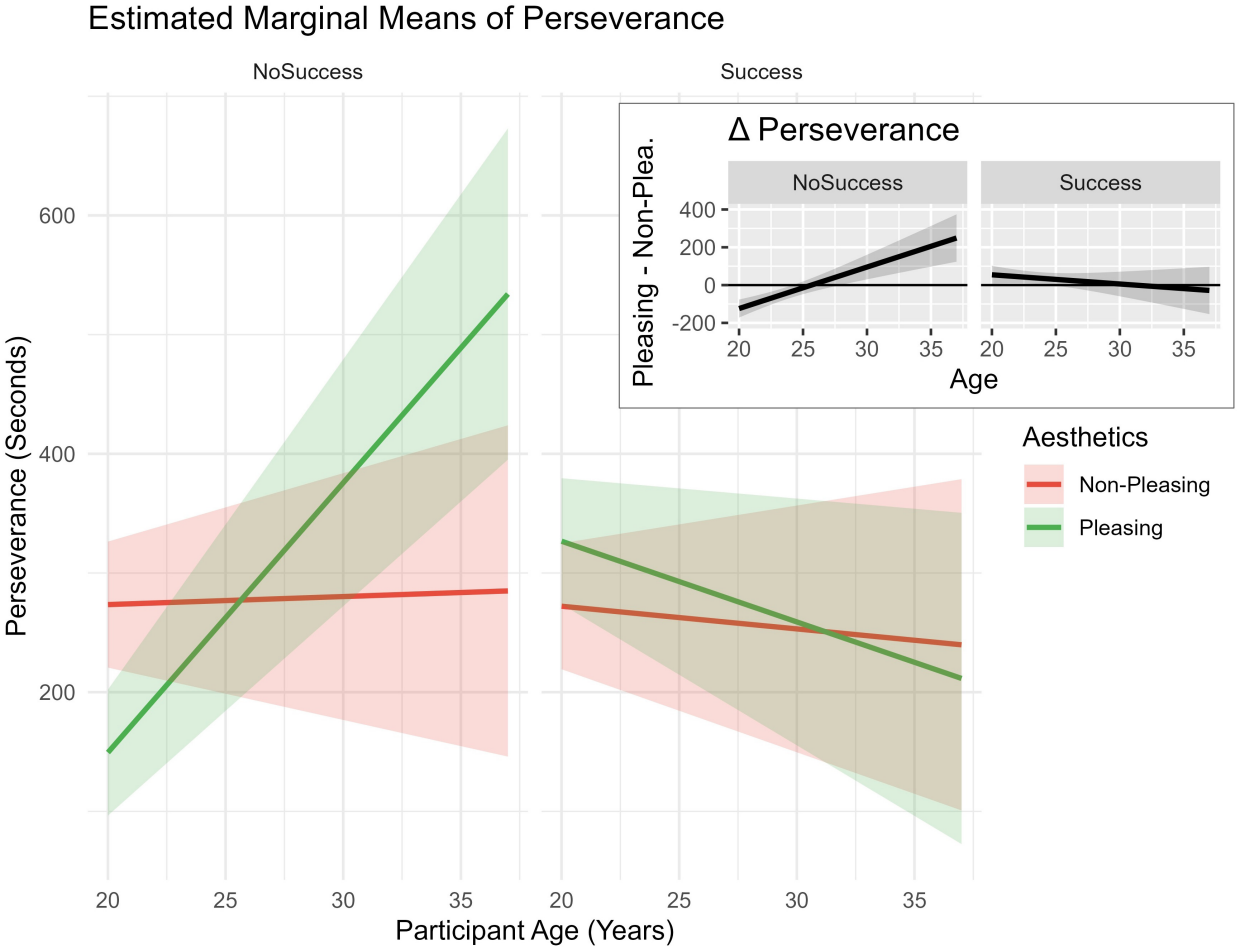
Estimated marginal means of perseverance predicted by “illusion of success,” “Aesthetics,” and “age.” Based on the mixed model. *Age* was centered with the sample mean. Shaded areas represent ±1 standard error of the mean (SEM).

The triple interaction between *illusion of success, aesthetics*, and *age* seemed to indicate a beneficial effect of *pleasing design* in the *no success* condition, especially for participants in their late twenties and early thirties (see Figure 5, Δ Perseverance). Interestingly, the effect seemed to be reversed for younger participants of the sample—*pleasing design* leading to lower *perseverance* times than *non-pleasing design* in the *no success* condition.

### 3.3 Motivation

For the analysis of participants' motivation, the standard model *[SIMS*_*Global*_ ~ *Aesthetics* ^*^ *IllusionOfSuccess* ^*^ *Age* + *(1|ID)]* did not provide a good fit. Accordingly, the next smallest was chosen in a data-driven way via stepwise exclusion of interaction and main effects (see Section 3.6 Statistical Analysis). The final model, *SIMS*_*Global*_ ~ *IllusionOfSuccess* + *(1|ID)*, exhibited good fit and differed significantly from model 0, $X_{\left(1,\;\text{N}=24\right)}^{2}$ = 4.54, *p* = 0.032. The analysis revealed a positive effect of the predictor *illusion of success*, indicating that participants in the *success* condition showed higher *motivation* scores, *b* = 0.19, *SE* = 0.09, *t*_(71)_ = 2.15, *p* = 0.035, $\eta_{\text{p}}^{2}$ = 0.061. No other effects were observed.

### 3.4 Workload

In order to capture the picture of *aesthetic* effects on *workload*, the following model was applied: *Workload*_*Global*_ ~ *Aesthetics* ^*^ *IllusionOfSuccess* ^*^ *Age* + *(1|ID)*. The model provided a good fit and differed significantly from model 0, $X_{\left(7,\text{N}=24\right)}^{2}$ = 43.62, *p* < 0.001. The respective analysis showed a negative effect of *illusion of success* signaling participants in the *success* conditions experienced lower workload; *b* = −17.33, *SE* = 3.61, *t*_(66)_ = −4.80, *p* < 0.001, $\eta_{\text{p}}^{2}$ = 0.441. No other effects were observed (all *p*s ≥ 0.144).

In addition to the overall score analysis, the subdimensions *mental demand* and *physical demand* were examined. The following *mental demand* model was created: *Workload*_*MentalDemand*_ ~ *Aesthetics* ^*^ *IllusionOfSuccess* ^*^ *Age* + *(1|ID)*. The full model showed a poor fit. Iterative, data-driven removal of interaction terms resulted in the model *Workload*_*MentalDemand*_ ~ *Aesthetics* + *IllusionOfSuccess* + *Age* + *(1|ID)*, aligning well with the data in comparison to model 0, $X_{\left(3,\text{N}=24\right)}^{2}$ = 30.78, *p* < 0.001. Results showed a negative effect of *illusion of success*; *b* = −21.25, *SE* = 3.56, *t*_(70)_ = −5.97, *p* < 0.001, $\eta_{\text{p}}^{2}$ = 0.337. No other effects were observed, all *p*s ≥ 0.313.

**Figure 6:**
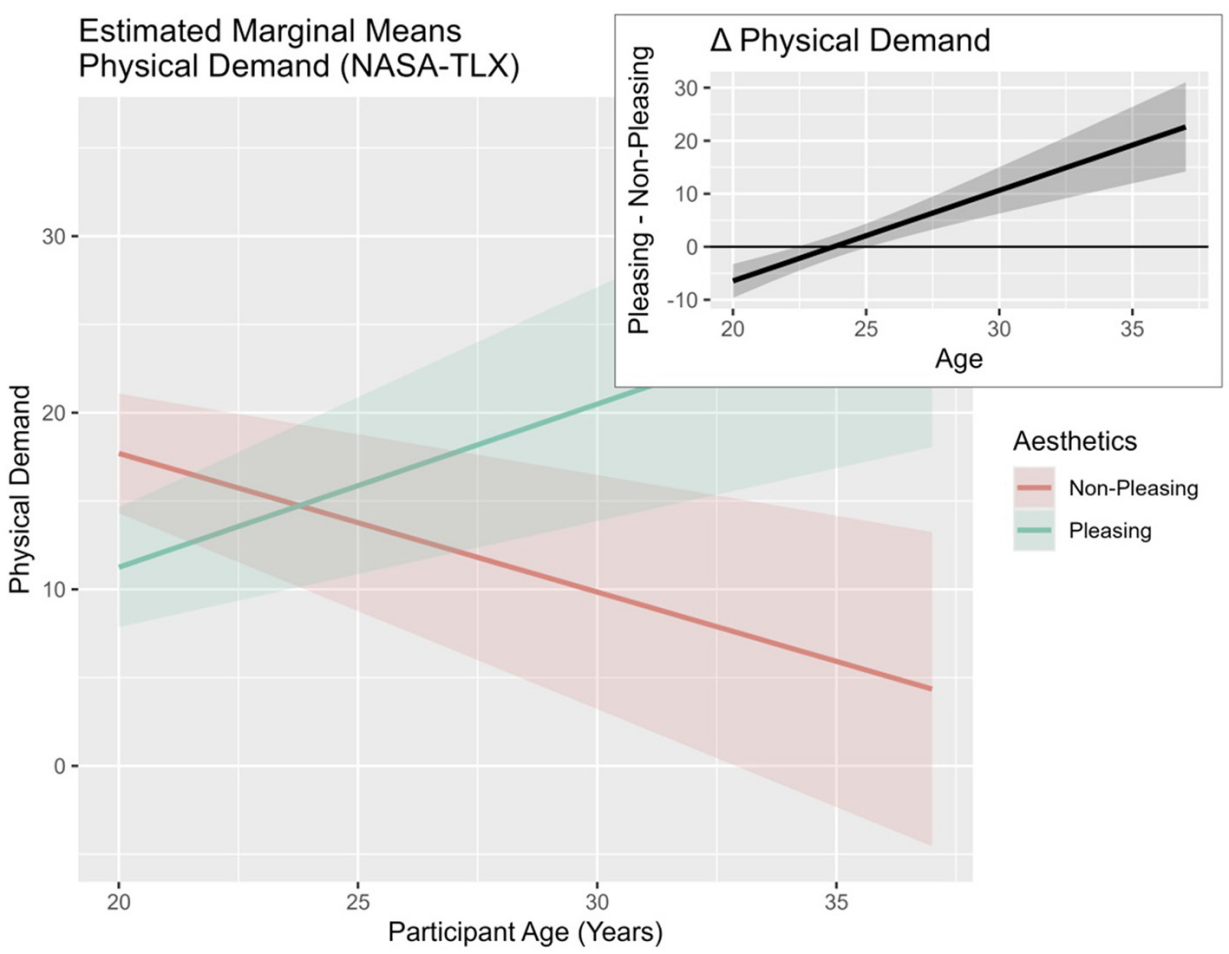
Estimated marginal means plot of double interaction effects on “physical demand” (NASA-TLX) predicted by “aesthetics” * “age.” Plot based on the fitted linear mixed-effects model. *Age* was centered with the sample mean. Shaded areas represent ±1 standard error of the mean (SEM).

With respect to *physical demand*, the applied model *PhysicalDemand* ~ *Aesthetics* ^*^ *IllusionOfSuccess* ^*^ *Age* + *(1|ID)* exhibited a good fit, contrasted with model 0, $X_{\left(7,\text{N}=24\right)}^{2}$ = 23.82, *p* = 0.001. No main effects were discovered. *Aesthetics* interacted with *age* (see Table 2), and participants in their late twenties and early thirties reacted with increased *physical demand* to the *pleasing design* condition (see Figure 6).

**Table 2:** Mixed-model analyses: effects of predictors “aesthetics,” “perception of success,” and “age” on physical demand.

**Predictor**	**Estimate**	**Std. Error**	***df***	***t-*value**	***p***	*** $\eta_{\text{p}}^{2}$ ***
(Intercept)	17.29	2.74	61.22	6.32	**< 0.001**	**–**
PleasDes	1.04	3.05	66.00	0.34	0.733	0.000
Succ	−5.21	3.05	66.00	−1.71	0.092	0.110
Age	−1.22	0.79	61.22	−1.55	0.125	0.001
PleasDes:Succ	−1.88	4.31	66.00	−0.44	0.665	0.003
PleasDes: Age	3.45	0.87	66.00	3.94	**< 0.001**	0.104
Succ: Age	0.87	0.87	66.00	0.10	0.322	0.029
PleasDes:Succ: Age	−3.48	1.24	66.00	−2.81	**0.007**	0.107

The table summarizes analyses of predictors of illusion of success (success vs. no success), aesthetics (pleasing design vs. non-pleasing design), and age (centered) on **physical demand**. PleasDes, pleasing design; Succ, success; df, degrees of freedom; p, probability of error.

**Figure 7:**
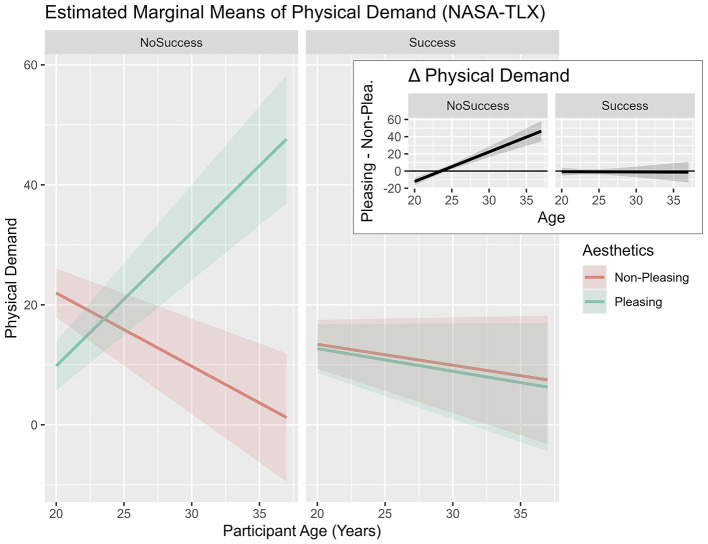
Estimated marginal means plot of triple interaction effects on “physical demand” (NASA-TLX) predicted by “aesthetics,” “illusion of success,” and “age.” Plot based on the fitted linear mixed-effects model. *Age* was centered with the sample mean. Shaded areas represent ±1 standard error of the mean (SEM).

In line with the double interaction results, an effect of the triple interaction between *aesthetics, illusion of success*, and *age* was found. Increased *physical demand* was experienced by participants in their late twenties and early thirties in the *no success* condition in reaction to a *pleasing design* in comparison to a *non-pleasing design* (see Figure 7). Moreover, younger participants experienced higher *physical demand when* interacting with the *non-pleasing* design in the *no success* condition.

### 3.5 EEG—alpha activity

**Figure 8:**
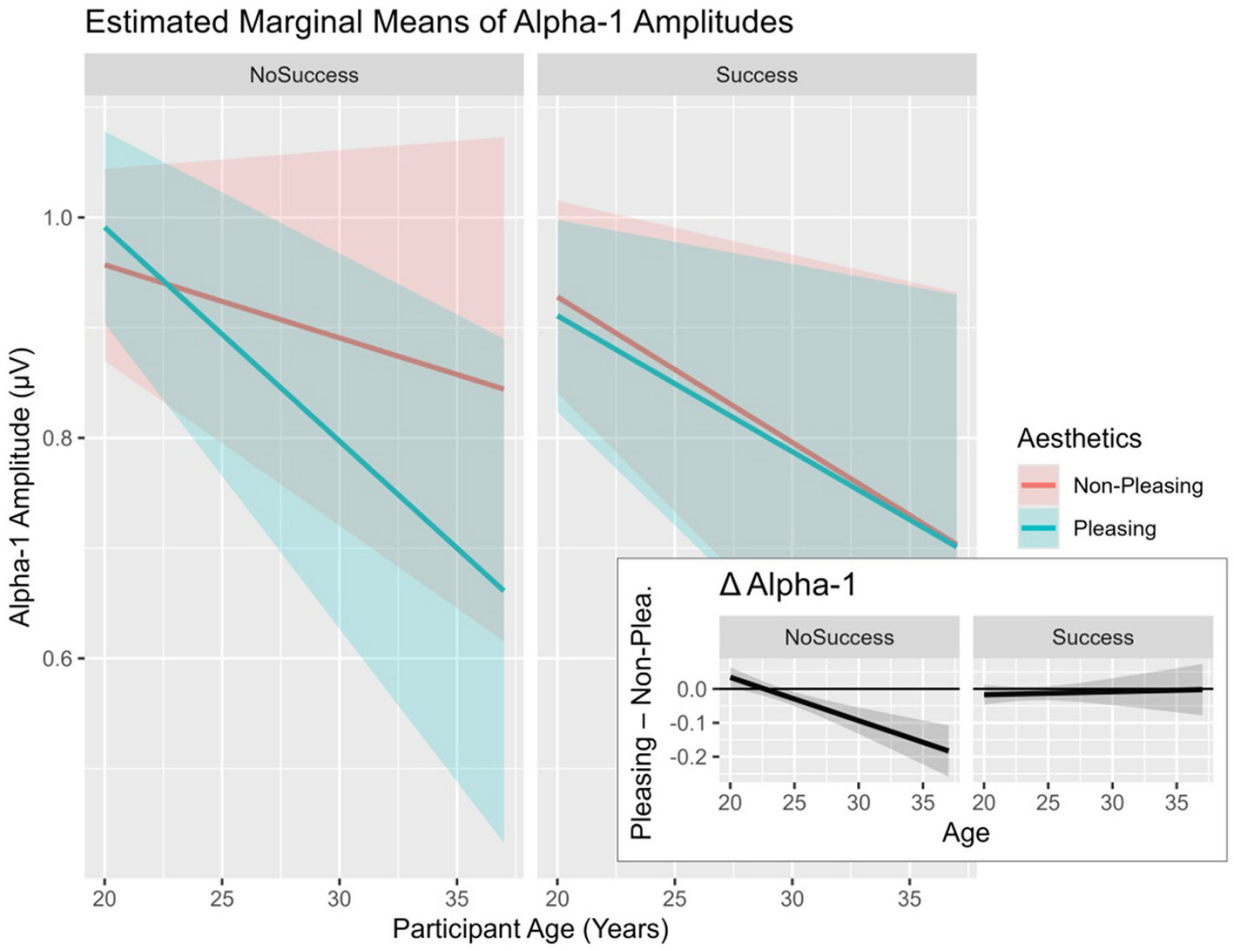
Alpha-1 Amplitudes in response to predictors “illusion of success,” “aesthetics,” and “age.” EEG sites averaged over the following sites: FP1, FP2, F3, F4, F7, and F8. *Age* was centered with the sample mean. Error bars indicating SEMs.

The mixed model to analyze EEG alpha activity in different conditions was defined as *Alpha-1* ~ *Aesthetics* ^*^ *IllusionOfSuccess* ^*^ *Age* + *(1|ID)*. The model provided a good fit relative to model 0, $X_{\left(7,\text{N}=24\right)}^{2}$ = 21.02, *p* = 0.004. A negative main effect of *illusion of success* was observed (see Table 3). In terms of the interaction between the *aesthetics* manipulation and *age*, an effect was found revealing that participants in their late twenties and early thirties experienced the *pleasing design* condition decreased *alpha-1* amplitudes in frontal areas (see Figure 8, Δ Alpha-1).

**Table 3:** Mixed-model analyses: effects of predictors “aesthetics,” “illusion of success” and “age” on alpha-1 amplitudes.

**Predictor**	**Estimate**	**Std. Error**	***df***	***t-*value**	***p***	*** $\eta_{\text{p}}^{2}$ ***
(Intercept)	0.931	0.059	24.07	15.896	**< 0.001**	**–**
PleasDes	−0.016	0.020	243.26	−0.785	0.433	0.004
Succ	−0.055	0.020	243.19	−2.719	**0.007**	0.055
Age	−0.007	0.017	23.94	−0.395	0.696	0.027
PleasDes:Succ	0.002	0.028	243.07	0.067	0.947	0.000
PleasDes: Age	−0.013	0.006	243.11	−2.280	**0.024**	0.009
Succ: Age	−0.007	0.006	243.04	−1.178	0.240	0.000
PleasDes:Succ: Age	0.014	0.008	243.05	1.730	0.085	0.012

The table summarizes the statistical analyses of predictors of illusion of success (success vs. no success), aesthetics (pleasing design vs. non-pleasing design), and age (centered) on alpha-1 amplitudes averaged over dlPFC, MFC, and OFC. Cut-off frequencies of the alpha-1 band were defined as an individual alpha peak (IAP) frequency and IAP-−2 Hz. PleasDes, pleasing design; Succ, success; df, degrees of freedom; p, probability of error.

Analog to *alpha-1* amplitudes, *alpha-2* amplitudes were analyzed. The mixed model *Alpha-2* ~ *Aesthetics* ^*^ *IllusionOfSuccess* ^*^ *Age* + *(1|ID)* was deployed. The fit was evaluated by comparison to model 0, $X_{\left(7,\text{N}=24\right)}^{2}$ = 16.87, *p* = 0.018. No (interaction) effects of the manipulations *Aesthetics, Illusion of Success*, or the *Age* variable were observed (*p*s ≥ 0.120).

### 3.6 Exploratory Bayesian network analysis

**Figure 9:**
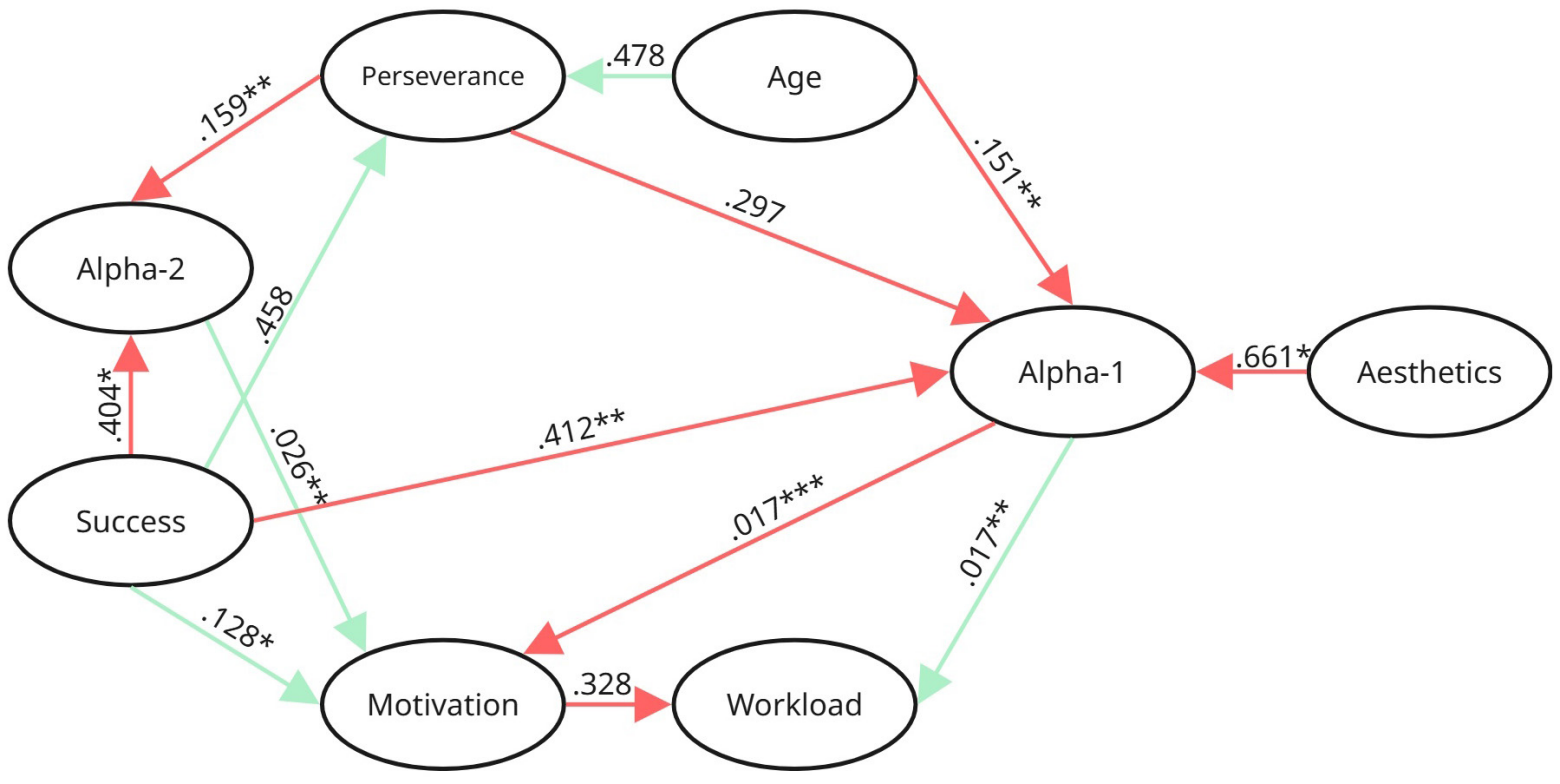
Exploratory Bayesian network analysis. The exploratory Bayesian network model represents data-driven directional associations. Edge labels indicate the strength of association based on pairwise Pearson correlations, green lines denoting positive associations and red lines denoting negative ones. Arcs are annotated based on their stability across 50′000 bootstrap replications (hill-climbing algorithm): *≥65% (tentative confidence), **≥80% (moderate confidence), ***≥95% (strong confidence) appearance rates.

An exploratory Bayesian network to identify directional associations among observed variables was created. The model structure was learned by applying a hill-climbing (HC) algorithm with directional constraints applied to the independent variables. *Aesthetics, Illusion of Success*, and *age* were treated as exogenous predictors, and incoming associations were blacklisted. Note, arcs in the model were annotated based on their stability across 50′000 bootstrap replications using HC structure learning (see Figure 9). Edges marked with ^*^ appeared in ≥65% of bootstrap samples (tentative evidence), ^**^ in ≥80% (moderate evidence), and ^***^ in ≥95% (strong evidence). According to Scutari (2010), the bootstrapping procedure provides an indication of confidence for each of the node connections.

As illustrated in Figure 9, *Aesthetics* shared a direct arc with *alpha-1* activity only (*r* = −0.66^*^) and indirectly influenced *workload* (*alpha-1 -*> *workload, r* = 0.02^**^) and *motivation* (*alpha-1 -*> *motivation, r* = −0.02^***^). *Illusion of success* showed a direct association with *perseverance* times (*r* = 0.46) as well as an association with *alpha-1* amplitudes (*r* = −0.41^**^) and *alpha-2* amplitudes (*r* = −0.40^*^). Additionally, there was a connection between *illusion of success* and *motivation* (*r* = 0.13^*^) that was established directly but also indirectly via the *alpha-1* (*alpha-1*-> *motivation, r* = −0.02^***^) and *alpha-2* (*alpha-2*-> motivation, *r* = 0.03^**^) pathway. Additionally, indirect connections were observed with *workload* through *motivation* (*motivation*-> *workload, r* = −0.33) and *alpha-1* (*alpha-1 -*> *workload, r* = 0.02^**^). Moreover, through *perseverance, illusion of success* further influenced *alpha-1* (*perseverance*-> *alpha-1, r* = −0.30) and *alpha-2* activity (*perseverance*-> *alpha-2, r* = −0.16^**^).

Participant *age* showed a direct connection with *perseverance* times (*r* = *0.4*8). Additionally, *age* was associated directly (*r* = −0.15^**^) and indirectly (*perseverance*-> *alpha-1, r* = −0.30) with *alpha-1* activity. Via *alpha-1, age* seemed to exhibit downstream effects on *workload* (*alpha-1 -*> *workload, r* = 0.02^**^) and *motivation* (*alpha-1*-> *motivation, r* = −0.02^***^). Through *perseverance* times, *age* also affected *alpha-2* amplitudes (*perseverance*-> *alpha-2, r* = −0.16^**^).

In order to evaluate the robustness of the network structure derived via HC search, an additional estimation of the model using the Tabu search algorithm (Glover, 1986), a well-established score-based structure learning procedure implemented in the bnlearn R package (Scutari, 2010). The resulting network was identical to the HC-derived structure, with perfect correspondence in all directed edges (true positives = 17; false positives = 0; false negatives = 0). This convergence suggests high stability and reliability of the learned network across alternative score-based optimization techniques.

## 4 Discussion

The main hypothesis of this study assumed that the positive influence of *aesthetics* on behavior would mainly occur when the task was difficult. Indeed, interaction effects between *aesthetics* and the *illusion of success* were observed regarding subjective and behavioral outcomes. In the context of the *perseverance* variable, a significant triple interaction effect was detected. More specifically, the inspection of the provided estimated marginal means plots (see Figure 5) shows increased *perseverance* times in late-twenties participants, especially in reaction to the *no success* condition when the *design* was *pleasing*. A similar pattern was observed in the context of *physical demand* (see Figure 7). Participants in their late twenties and early thirties persevered longer at the task at hand and, consequently, experienced higher *physical demand*. These findings seem to be in accordance with our expectation—*pleasing design* might have influenced participants in their late twenties to persevere, when they were experiencing *no success*. This finding appears to be consistent with studies in the context of *aesthetics* and performance, indicating “when the going gets tough” (Reppa and McDougall, 2015)—i.e., not being successful during NFB, the beautiful get going” (Reppa and McDougall, 2015) or *pleasing design* leads to increased *perseverance* times.

Note that the reported interaction effects only affected participants in their late twenties and early thirties in that way, which might be due to developmental processes of the PFC (Ferguson et al., 2021) influencing tasks requiring self-control (i.e., not stopping the NFB, although it might be hard to continue). This interpretation aligns with continued pruning processes until the mid-twenties (Kolk and Rakic, 2022). Additionally, the finding of increased *perseverance* in reaction to *pleasing design* in the *no success* condition seems to be in line with effects relating to societal changes that different participants in the sample have experienced differently. Younger participants of the study have grown up with pronounced interactions with smartphones, which were commonly adopted from 2010 to 2015 (Silver, 2019). During that time, early-twenty participants were around 10 years old, while late-twenty participants were around 20 years of age. This difference arguably led to a differential exposure to smartphone use, which has been demonstrated to affect the neurodevelopment of reward processing and cognitive control (Marciano et al., 2021).

Thus, smartphone use and adoption might have affected *perseverance* in study participants differentially in interaction with *age*. This interpretation is coherent with the fact that the studies reporting effect patterns as hypothesized in former studies (“when the going gets tough, the beautiful get going,” Reppa and McDougall, 2015; Moshagen et al., 2009) were conducted before 2015 with participants who had grown up in a digital environment more similar to the context of late-twenty/early-thirty participants in the current study.

Another indicator for the perspective of executive functions influencing outcomes in the study at hand comes from the analysis of neurophysiological outcomes. More specifically, *alpha-1* was affected by the interaction of *pleasing* design and *age*, showing decreased *alpha-1* amplitudes in older participants in reaction to *pleasing* design (see Figure 8, Δ Alpha-1). As alpha at frontal locations seems to play a prominent role in the study at hand, the result is congruent with studies underlining the important role of alpha dynamics in the context of executive functioning (Vera et al., 2024) in frontal locations. Additionally, a possible interpretation of the effect may lie in linking the observed EEG pattern to existing literature on neuroaesthetic effects, implying increased fMRI activity in frontal areas in reaction to *pleasing design* stimuli (Kawabata and Zeki, 2004; Jacobsen et al., 2006; Jacobs et al., 2012; Vartanian and Goel, 2004). As studies support the notion of an inverse relationship between fMRI BOLD signal and EEG alpha activity (Laufs et al., 2003), lowered *alpha-1* levels in frontal areas could speak in favor of increased activity or blood flow in frontal areas in the current study in late-20/early 30 participants in interaction with the *pleasing design*. Note that a corresponding node connection was also obtained from the exploratory Bayesian network analysis—higher *pleasing design* leading to lower *alpha-1* levels.

While the neurophysiological and behavioral results supported the notion of the expected beneficial effects of *pleasing design* in conditions of *no success* with some inconsistencies, the subjective questionnaire data did not follow the conjectured effects pattern. In the context of *workload* in interaction with *pleasing* and *age*, an intensifying *physical demand* (see Figure 7) was uncovered in participants in their late twenties and early thirties. The finding might be interpreted as a result of the emphasized perseverance behaviors of participants in their late twenties and early thirties (see Figure 5). Participants potentially exert greater effort in response to the *pleasing design*. It seems plausible that the facilitating effect of *aesthetics* on *perseverance* is unlocked by fully mature executive functioning. This finding supports the notion that younger participants of this study might not have profited from the expected *aesthetic* effects because of continued prefrontal cortex development until the mid-twenties (Ferguson et al., 2021; Kolk and Rakic, 2022). Apart from general prefrontal cortex development and executive function maturation, additional environmental factors in terms of smartphone use and social media use might have affected the tested sample differentially.

Regarding participant *motivation*, only the effect of the *illusion of success* was significant. Unexpectedly, no interaction with the *aesthetic* manipulation was observed, and in the exploratory Bayesian model, the incoming arc from the *illusion of success* seems to confirm the effect of the *illusion of success* variable. The lack of hypothesis-confirming motivational effects could be due to the small sample size or the nature of the study, which tested healthy participants in the university context.

From the perspective of Self-Determination Theory (SDT; Ryan and Deci, 2017), the missing effect of *aesthetics* could be attributed to the limited personal relevance of the study contents and the simultaneous presence of a small extrinsic incentive (i.e., course credit as compensation for the participation). Traditionally, it is assumed that extrinsic rewards undermine intrinsic motivational effects and lead to lower interest (Deci et al., 1999). The provision of an external reward in the study at hand, without ensuring individual relevance of the study contents for the participants, might explain the missing motivational effects. More recent literature argues for a synergistic effect of intrinsic motivation and extrinsic rewards (Cerasoli et al., 2014), and from that point of view, the provided incentive might have been insufficient to activate sizable motivational effects in the current study.

The limitation of the low motivational relevance of the study contents for the participants could be solved in future studies by considering patient populations. As Haugg et al. (2021) argue, patients exhibit better self-regulatory performance than other participant populations. Using a clinical sample (e.g., Tinnitus sufferers) as a test population might lead to greater engagement and, concurrently, stronger effects of the manipulation on main outcomes. In the current study, we chose to exclude the patient population because of ethical issues that arise from including patient populations in an S-NFB study. An ethically viable S-NFB study would have to comprise a contingent NFB arm, increasing the resources spent substantially. The allocation of additional resources may now be justified, given that initial evidence for design-related effects has been demonstrated in the present study.

The added statistical power in future studies seems crucial to evaluate the effects of critical covariates. One exemplary third variable relates to recent findings indicating that hormonal effects interact with workload, affecting perseverance, performance, and cognitive task outcomes measuring executive functions (Bradshaw et al., 2020; Xu et al., 2022). Although effect sizes for potential confounds remain to be determined, our within-subjects design minimizes between-subjects covariation effects by applying a within-subject design and collecting each participant's data in one single test session.

In sum, the present study investigated whether the *aesthetics* of an S-NFB visualization, as well as the *illusion of success*, improve the S-NFB experience. Results seemed to align with the main hypotheses in terms of *perseverance* and, in part, with EEG *alpha* activity, and interacted with participant age. This study presents the first evidence that *pleasing design* indeed influences participant outcomes in the context of NFB, and further research, including verum NFB training in the patient population, is needed to confirm the initial results observed in the study at hand. Furthermore, the findings appear to highlight the relevance of potential technology adoption effects in younger individuals—an effect previously observed in domains such as higher education (Baert et al., 2020) and further supported by evidence linking excessive smartphone use to diminished executive functioning (Henemann et al., 2023).

## Data Availability

All relevant data are available from: https://doi.org/10.5281/zenodo.15132298.

## Author's note

Supplementary materials are provided via Zenodo: Naas A, Rohrbach S, Shabestari P, Neff P, Sonderegger A. Supplementary Materials – Aesthetics, illusion of success and age interactions: disentangling effects in the closed-loop design with sham neurofeedback training. Geneva, Switzerland: Zenodo; 2025. Available from: https://doi.org/10.5281/zenodo.15132298.
